# Parliament and the Profession

**Published:** 1917-03-03

**Authors:** 


### PARLIAMENT AND THE PROFESSION.

### Discharged Soldiers with Tuberculosis.

Sir E. Cornwall informed Mr. Hogge that under special arrangements made by the Admiralty and War Office with various Insurance Commissions beds have been provided for nearly 5,000 discharged sailors and soldiers suffering from tuberculosis. In addition, under the ordinary arrangements beds have been provided for a considerable number of discharged soldiers. Sir E. Cornwall further stated that these admissions have the effect of increasing the waiting-list of civilians.

### Special Grant for Tuberculosis.

On February 19 Parliament authorised a supplementary grant of £27,000 towards the cost of sanatorium benefit under the National Insurance Act, 1911, and of the treatment of tuberculosis generally. This extra sum is to be allocated to the Local Government Board for Scotland, owing to the expenditure of local authorities being greater than that anticipated. Mr. Booth, in connection with this vote, raised a discussion upon the general administration of the tuberculosis benefit.

### Medical Notes of Wounded Men.

Mr. Macpherson, who was asked by Mr. Snowden if wounded men from Flanders could be accompanied by their medical notes to avoid delay on arrival in England, stated that each man has attached to his coat a card giving particulars of the case. Improvements of this card are now being carried out, and on it a fuller history will be given.

### Manipulative Surgery.

Several inquiries have been put to the Under-Secretary of State for War with a view to reopen the question of the employment of unqualified practitioners to perform manipulative surgery in military hospitals. Mr. Macpherson has not added anything of importance to his statement on the subject reported in The Hospital on February 24, p. iv.

